# Regional Cultures and the Psychological Geography of Switzerland: Person–Environment–Fit in Personality Predicts Subjective Wellbeing

**DOI:** 10.3389/fpsyg.2018.00517

**Published:** 2018-04-16

**Authors:** Friedrich M. Götz, Tobias Ebert, Peter J. Rentfrow

**Affiliations:** ^1^Department of Psychology, University of Cambridge, Cambridge, United Kingdom; ^2^Mannheim Centre for European Social Research, University of Mannheim, Mannheim, Germany

**Keywords:** geographical psychology, regional cultures, person–environment–fit, big five personality traits, subjective wellbeing, MLM, Swiss Household Panel, research methodology

## Abstract

The present study extended traditional nation-based research on person–culture–fit to the regional level. First, we examined the geographical distribution of Big Five personality traits in Switzerland. Across the 26 Swiss cantons, unique patterns were observed for all traits. For Extraversion and Neuroticism clear language divides emerged between the French- and Italian-speaking South-West vs. the German-speaking North-East. Second, multilevel modeling demonstrated that person–environment–fit in Big Five, composed of elevation (i.e., mean differences between individual profile and cantonal profile), scatter (differences in mean variances) and shape (Pearson correlations between individual and cantonal profiles across all traits; Furr, 2008, 2010), predicted the development of subjective wellbeing (i.e., life satisfaction, satisfaction with personal relationships, positive affect, negative affect) over a period of 4 years. Unexpectedly, while the effects of shape were in line with the person–environment–fit hypothesis (better fit predicted higher subjective wellbeing), the effects of scatter showed the opposite pattern, while null findings were observed for elevation. Across a series of robustness checks, the patterns for shape and elevation were consistently replicated. While that was mostly the case for scatter as well, the effects of scatter appeared to be somewhat less robust and more sensitive to the specific way fit was modeled when predicting certain outcomes (negative affect, positive affect). Distinguishing between supplementary and complementary fit may help to reconcile these findings and future research should explore whether and if so under which conditions these concepts may be applicable to the respective facets of person–culture–fit.

## Introduction

The world we live in offers an overwhelming economic, political, cultural, social and climatic diversity (Rentfrow, 2014a) and one will hardly ever find two places that are exactly alike. How then do the characteristics of our environments shape the behavior and attitudes of their inhabitants? Without a doubt, culture, defined as the part of the environment made by humans (Oyserman, 2017), plays a major role. Whilst the measurement of culture is not trivial, a common way to capture cultural differences is the comparison of aggregated culture-level personality traits, which are assumed to reflect the central tendencies of the members of that respective culture (Rentfrow et al., 2008; McCann, 2010, 2011; Obschonka, 2017). Using the most widely endorsed taxonomy of personality traits (Kitayama et al., 2006; Mõttus et al., 2017), i.e., the Five-Factor Model of personality (Tupes and Christal, 1992; McCrae and Costa, 2013; FFM), researchers have compiled ample evidence that cultures differ significantly along the Big Five personality traits (i.e., Agreeableness, Conscientiousness, Extraversion, Neuroticism, Openness; McCrae, 2001; Allik and McCrae, 2004; McCrae et al., 2005; Schmitt et al., 2007). Drawing on these findings, Fulmer et al. (2010) proposed the person–culture match hypothesis, arguing that the individual fit between a person and their surrounding culture should positively predict psychological wellbeing. Indeed, although the person–culture–fit literature is still sparse (Oyserman, 2017), the evidence available suggests that a high person–culture–fit is associated with increased psychological (Lu, 2006; Fulmer et al., 2010; De Leersnyder et al., 2015) as well as relational wellbeing (De Leersnyder et al., 2014), higher life satisfaction (Musiol and Boehnke, 2013), adherence to healthy diets (Levine et al., 2016), and fewer relationship problems (Friedman et al., 2010). Moreover, a good fit between the cultural environment and one's religiosity (Bleidorn et al., 2016), agency (vs. communion; Gebauer et al., 2013), avoidance focus (vs. proactivity; Diener et al., 2003), predicts self-esteem and psychological adjustment.

Of note, it has been shown that cultural fit, rather than scoring high on desirable personality traits *per se* predicts these beneficial outcomes (Denissen et al., 2017). Yet, as person-culture–fit is evolving into a key concept in personality psychology (Cohen and Varnum, 2016; Rentfrow and Jokela, 2017), scholars have called into question whether a traditional, nation-based understanding of culture provides the most adequate frame of reference for this line of research (e.g., Musiol and Boehnke, 2013; Cohen and Varnum, 2016; Rentfrow and Jokela, 2016). More to the point, researchers have argued that psychology has largely overlooked the cultural variations that exist within countries (Cohen, 2009; Harrington and Gelfand, 2014). Moving away from the narrative of nations as homogeneous, monolithic cultures (Plaut et al., 2012; Cohen and Varnum, 2016), recent years have witnessed the rise of geographical psychology (Rentfrow et al., 2008; Florida and Mellander, 2014). Acknowledging the importance of intra-national cultural diversity (Cohen, 2009; Nettle and Colléony, 2014; Rentfrow and Jokela, 2016), geographical psychology seeks to understand regional differences in psychological characteristics by means of Big Data analysis (Oishi, 2015; Obschonka, 2017). This is of great interest as the psychological characteristics that dominate geographical areas may ultimately drive and sustain the emergence of regional cultures and correspondingly translate into distinct economic and social climates (Rentfrow et al., 2013; Obschonka et al., 2016). In other words, the study of the geographical distribution of psychological constructs may foster a more nuanced understanding of regional cultures that are nested within national cultures (Conway et al., 2001).

Over the course of the past decade, personality differences within nations have received strong empirical support (Rentfrow, 2014b). While most research originates from the United States (Levine et al., 1994, 2008; Conway et al., 2001; Florida, 2002, 2014; Park et al., 2006; Rentfrow et al., 2008; Park and Peterson, 2010; Rentfrow, 2010; Harrington and Gelfand, 2014; Graham et al., 2016; Bach et al., 2017; Chopik and Motyl, 2017; Findley and Brown, 2017), similar patterns have been observed in other countries such as Italy (Camperio Ciani et al., 2007; Camperio Ciani and Capiluppi, 2011), Japan (Kitayama et al., 2006), Russia (Allik et al., 2009), the UK (Rentfrow et al., 2015), and China (Wei et al., 2017). Attesting to their real-world importance, regional personality differences have been linked to a host of critical outcomes, such as personal wellbeing (Rentfrow et al., 2009), emotional health (McCann, 2011), smoking prevalence (McCann, 2010), suicide rates (Voracek, 2009), drug use, discrimination, incarceration rates (Harrington and Gelfand, 2014), entrepreneurial activity (Obschonka et al., 2013, 2015), patent production (Lee, 2017) and economic resilience (Obschonka et al., 2016).

In addition to macro-level outcomes, individual-level outcomes appear to be equally affected by regional cultures (Rentfrow and Jokela, 2017). As an extension of the person–culture match hypothesis (Fulmer et al., 2010) the person–environment–fit hypothesis operates on a more geographically fine-grained level, stipulating that living in an environment that matches one's own profile provides better chances to satisfy one's psychological and physical needs (Rentfrow et al., 2008) and is associated with higher life satisfaction (Jokela et al., 2015; Oishi, 2015; Oishi et al., 2015). Consistent with the person–environment–fit hypothesis, regional fit in political orientation has been shown to bear on subjective wellbeing (Stavrova and Luhmann, 2016). Moreover, the same patterns were replicated on an even more granular level of analysis, pointing to the multi-faceted, hierarchical nature of culture (Cohen, 2009; Stavrova and Luhmann, 2016; Oyserman, 2017). An Australian study found that high congruence between participants' personalities and where they lived was linked to better mood (Murray et al., 2005). Similarly, person–city–fit had a positive effect on entrepreneurial success (Zhou et al., 2017) and self-esteem (Bleidorn et al., 2016). In a longitudinal study at the University of Berkeley, a better match between students and the university culture was correlated with higher levels of personality consistency and self-esteem as well as lower levels of Agreeableness and Neuroticism (Roberts and Robins, 2004). Greater person–university–culture–fit has also been linked to lower cortisol levels (Stephens et al., 2012) and enhanced wellbeing among ethnic minorities (Gloria et al., 2005). Examining person–environment–fit across 216 postal districts in the London metropolitan area, Jokela et al. (2015) observed that links between Openness, Agreeableness and Conscientiousness with life satisfaction were contingent on the characteristics of one's district. Thus, while the evidence presented above underscores the relevance and meaningfulness of fit, one of the main issues of fit research lies in its operationalisation.

In recent years, many different approaches to the measurement of fit have been employed (Phillips et al., 2010), ranging from intraclass correlation coefficients (ICC; Fulmer et al., 2010), sums of absolute differences (Noë et al., 2016), as well as sums of squared differences (Obschonka et al., 2013), and squared Euclidian distances (Musiol and Boehnke, 2013) to response surface analysis (RSA) techniques (Bleidorn et al., 2016; Denissen et al., 2017; Zhou et al., 2017). Undeniably, all of these methods have their merits. Yet, in the present research, we have decided to follow Furr (2010; Wood and Furr, 2016), who makes a compelling case against the use of an omnibus index of profile similarity and advocates a differentiated approach instead. As—to our knowledge—no prior research in the fit literature has adopted such a holistic approach, our main motivation was to zoom in on the processes that determine good fit and investigate how they act in concert. Consequently, following Furr's recommendations, we calculated three fit indices (i.e., shape, elevation, and scatter), tapping into different facets, to jointly capture as much of the fit concept as possible.

Beyond measurement issues, scholars in the field have repeatedly urged the scientific community to extend its research efforts to new countries (Jokela et al., 2015; Bleidorn et al., 2016) and gain a more in-depth understanding of person–environment–fit (Rentfrow and Jokela, 2016). On a methodological note, the need for longitudinal data (Roberts and Robins, 2004; Motyl et al., 2014; Oishi, 2014; Delvaux et al., 2015; Jokela et al., 2015; Rentfrow and Jokela, 2016, 2017; Stavrova and Luhmann, 2016) and representative samples (Rentfrow et al., 2008; Jokela et al., 2015; Bleidorn et al., 2016) has been stressed.

In order to answer these calls, the present study draws from the main dataset of the Swiss Household Panel (SHP), a national panel study that collects annual data on a large, stratified, random sample of Swiss residents (Budowski et al., 2001), thus tracking a large cohort of individual participants over time. Aside from the availability of the rich dataset from the SHP, Switzerland represents an especially fascinating and well-suited case for the study of regional cultures. Offering its 26 cantons an exceptional degree of political, fiscal and cultural autonomy (Stojanovic, 2008), it is one of the most decentralized countries in the world (Linder and Vatter, 2001).

When the Swiss Confederation was founded in 1848, a Swiss society hardly existed (Linder and Vatter, 2001). Instead, the constitution attempted to unite the cantons that were deeply divided by four languages, two Christian religions and different ethnicities (Linder and Vatter, 2001). Consequently, the principle of federalism has been a defining element in the Swiss political philosophy (Linder and Vatter, 2001) and collective mindset (Crivelli et al., 2006) from the very beginning. Although some of the cleavages that characterized the Swiss Confederation in its early days have somewhat faded (Linder and Vatter, 2001), there are still large regional differences in present-day Switzerland. Amongst others, the cantons vary widely in population [ranging from 16,000 (Appenzell Inner Rohodes) to 1,466,424 (Zurich; Swiss Federal Statistics Office, 2017b), GDP per capita (Uri: 51,332 CHF; Basel-Stadt: 163,632 CHF, Swiss Federal Statistics Office, 2017a), urbanity, immigration (percentage of foreigners: 10.9% (Appenzell Inner Rohdes), 40.7% (Geneva; Swiss Federal Statistics Office, 2017b)], access to higher education (Linder and Vatter, 2001), extent of direct democratic rights (Frey and Stutzer, 2000; Linder and Vatter, 2001), egalitarianism (Linder and Vatter, 2001) and tax structures (Feld and Kirchgässner, 2002). Meanwhile, other historical fault lines, such as religion (Roman Catholics: 37.9%, Reformed Protestants: 23.5%, no religious affiliation: 23.0%; Swiss Federal Statistics Office, 2017a) and predominant language (German: 64.5%, French: 22.7%, Italian: 8.4%, Romansh: 0.5%; Swiss Federal Statistics Office, 2017a) are still pronounced and continue to influence important outcomes. For instance, variation in direct democracy on the cantonal level has been shown to predict happiness (Frey and Stutzer, 2000), while regional differences in religion have been associated with suicide rates (Spoerri et al., 2010; Torgler and Schaltegger, 2014), both of which suggest an influence of regional differences in Switzerland on quality of life.

Against this backdrop, the current research sought to address two issues. First, as an extension of previous research (Rentfrow et al., 2008, 2015; Jokela et al., 2015), the present work mapped the geographical distribution of Big Five personality traits across the 26 cantons of Switzerland.

Second, multi-level modeling (MLM) was used to investigate whether person–environment–fit was associated with subjective well-being. Given its manifold downstream consequences, e.g., for health, longevity, social relationships and fertility, subjective wellbeing has evolved into a vibrant field (Diener, 2012; Diener et al., 2017), which is also reflected in a growing area of research at the nexus of culture fit and subjective wellbeing (e.g., Sagiv and Schwartz, 2000; Plaut et al., 2012; De Leersnyder et al., 2015; Oishi and Gilbert, 2016). However, the bulk of this work has been carried out at the national level (Rentfrow et al., 2009), failing to leverage the explanatory potential of fit with regional cultures. Moreover, despite the fact that it is known that subjective wellbeing is actually a multifaceted construct that is best measured by assessing its components (i.e., positive affect, negative affect, life satisfaction) in tandem (Diener, 2012; Diener et al., 2017), prior research in the field has oftentimes looked at single facets in isolation (e.g., Rentfrow et al., 2009; Musiol and Boehnke, 2013; Jokela et al., 2015). Therefore, we adopted a holistic approach. In addition to the three aforementioned components, we included satisfaction with personal relationships as a fourth outcome, reasoning that person–culture–fit first and foremost manifests itself as a social phenomenon. Previous research points to the suitability of relationship satisfaction as index of subjective wellbeing (Diener et al., 2017) and provides evidence that it is different from psychological wellbeing *per se* and thus measures a distinct construct in its own right (De Leersnyder et al., 2014, 2015). Taken together, we expected high person–environment–fit (i.e., low elevation, high shape, low scatter) to be predictive of elevated subjective wellbeing.

## Materials and methods

### Participants

Data from wave 11 (2009) to wave 17 (2015) of the SHP (described below) were used. Given the data collection conventions of the SHP, inter alia, the dataset contains minors from 14 to 17 years of age. Recognizing that personality is usually not yet fully developed and rather prone to drastic changes during one's youth (Soto et al., 2011), we excluded minors of <18 years of age from all analyses. Furthermore, only participants that had completed the Big Five measure were deemed eligible for analysis. Hence, our final sample consisted of 7,767 participants, of whom 44.71% were male and 55.29% were female. Participants had been monitored annually through the SHP. On average, participants were 46.8 years old (*SD* = 19.16), which is consistent with prior research if one bears in mind that minors were not included in the current study (e.g., Anusic et al., 2012). Regarding education status, 29.56% had at least graduated from high school and 16.4% had pursued a university-level education of some sort.

### Swiss household panel

Launched in 1999, the SHP is an on-going, large-scale, nationally representative, longitudinal study, whose principal aim is to act as a barometer of social change, monitor cultural dynamics and provide high-quality quantitative data for the social sciences (Budowski et al., 2001; Tillmann et al., 2016; Voorpostel et al., 2016). Attrition is generally comparable to similar panel studies such as the British Household Panel and the European Community Household Panel (Lipps, 2007). Thanks to two refreshment samples, recruited in 2004 and 2013 as of 2015, 16,348 Swiss residents participate in the panel (Tillmann et al., 2016; Voorpostel et al., 2016).

### Ethics and informed consent

In accordance with international ethical standards of research on humans, the SHP was approved by the Research Commission of the University of Neuchâtel (Zimmermann-Sloutskis et al., 2010). During every data collection wave, each household in the panel receives a letter containing extensive information about the SHP and its aims. Moreover, the length and content of the interviews are specified and the confidentiality, anonymity and exclusive use of the data for scientific research purposes are explained in detail. Shortly afterward, all households are contacted via telephone by the survey institute that conducts the data collection, and each household member can freely consent to participation or refuse it (Zimmermann-Sloutskis et al., 2010).

The SHP data are freely available to scientists who sign a contract with the Swiss Centre of Expertise in the Social Sciences (FORS), which oversees the SHP, agreeing to legal and ethical conditions that apply to the use of the data for research purposes (Tillmann et al., 2016). As part of that protocol, the scientists who wish to obtain access to the SHP data are required to offer an in-depth account of their research project and need to explain how they intend to use the SHP data. If the application is approved by FORS, access to the data is granted online via a personalized link to a secure data repository, where the SHP data is stored. The present work complied with all these stages and has satisfied the ethical and legal standards of FORS which has henceforth provided the researchers with the requested SHP data, thereby granting explicit permission to carry out the current research in its present form.

### Measures

#### Independent variables

##### Age and sex

Throughout the panel, participants were asked to report their age and sex at every data collection.

##### Education status

In every wave, participants were asked to select the status that corresponded to the highest level of education that they had achieved at the time of data collection, from a list of qualifications. The list consisted of 17 categories ranging from 0 = “incomplete compulsory school” to 16 = “Ph.D.” As is customary in psychological research, the 17 categories were regrouped into three broader categories, reflecting attainment of primary, secondary and tertiary education, respectively (1 = “less than graduated from high school”; 2 = “at least graduated from high school”; 3 = “at least some university-level education”).

##### Personality

Individual personality was measured employing the 10-item Big Five Inventory short scale (BFI-10; Rammstedt and John, 2007) derived from the BFI-44 (see John and Srivastava, 1999 for the original items and John et al., 2008 for evidence of reliability and validity). Unlike other variables in the SHP, the BFI was only administered once per person, during the earliest wave possible. Henceforth, whereas the majority of participants of the SHP completed the BFI when it was first used in 2009 (wave 11), others that entered the panel later on or missed the data collection in wave 11 did so in 2010 (wave 12) or 2011 (wave 13), respectively. In any case, respondents were asked to report their level of agreement with a series of self-descriptions on an 11-point scale, anchored at 0 = “disagree strongly” and 10 = “agree strongly.” Each trait was assessed by two items, comprising the following statements: “I see myself as someone who…”; Agreeableness: “is generally trusting,” “tends to find fault with others” (reversed), Conscientiousness: “does a thorough job,” “tends to be lazy” (reversed), Extraversion: “is outgoing, sociable,” “is reserved” (reversed), Neuroticism: “gets nervous easily,” “is relaxed, handles stress well” (reversed), Openness to experience: “has an active imagination,” “has artistic interests.” As is common for short scales that attempt to reflect conceptual breadth (Cronbach, 1951), inter-item correlations were *r* = 0.08 (agreeableness), *r* = 0.26 (conscientiousness), *r* = 0.36 (extraversion), *r* = 0.36 (neuroticism), and *r* = 0.21 (openness to experience), which is consistent with prior published research featuring personality data from the SHP (Anusic et al., 2012; Furler et al., 2013) and—with the exception of Agreeableness—general recommendations for psychometric evaluations (Clark and Watson, 1995). Moreover, the BFI-10 has been shown to correlate highly with the original BFI-44, capturing 70% of the full BFI variance, while retaining 85% of the BFI-44's test-retest reliability with virtually unchanged discriminant and structural validity (Rammstedt and John, 2007).

#### Dependent variables

##### Life satisfaction

A single-item measure of life satisfaction, the cognitive component of subjective wellbeing (Diener et al., 2017), was administered throughout all panel waves. The respective item read: “In general, how satisfied are you with your life, if 0 means “not at all satisfied” and 10 means “completely satisfied”?” Empirical evidence has been compiled to demonstrate the robustness and scientific suitability of this measure (Lucas and Donnellan, 2012; Cheung and Lucas, 2014) and it has been often used in SHP-based research (Dorn et al., 2007; Steiner et al., 2010; Furler et al., 2013; Anusic et al., 2014).

##### Negative affect

A measure of negative affect was administered during every panel wave, using the following item: “Do you often have negative feelings such as having the blues, being desperate, suffering from anxiety or depression, if 0 means “never” and 10 “always”?”

##### Positive affect

Analogously, a parallel question was asked, serving to assess positive affect, reading as follows: “Are you often full of strength, energy and optimism, if 0 means “never” and 10 “always”?”

##### Satisfaction with personal relationships

Satisfaction with personal relationships was continuously measured through the following question: “How satisfied are you with your personal, social and family relationships, if 0 means “not at all satisfied” and 10 “completely satisfied”?”

### Derivation of fit indices

The three fit indices were calculated in accordance with Furr (2008, 2010). In a first step, the cantonal Big Five profiles were calculated by aggregating the scores of their inhabitants, in line with prior research (Hofstede and McCrae, 2004; Gebauer et al., 2012, 2013; Rentfrow et al., 2013; De Leersnyder et al., 2014; Bleidorn et al., 2016).

Subsequently, elevation was assessed by subtracting the mean value of the individual profile, averaged across all Big Five traits, from the mean value of the individual's respective canton of residence, also averaged across all Big Five traits (Furr, 2008, 2010). Hence, elevation captures the average deviation of an individual profile, such as, the Big Five profile of a resident of the canton of Zurich from a normative profile, in this case the aggregate Big Five profile of the canton of Zurich. Absolute values were used to ensure that the resulting elevation values would always be positive.

To determine scatter, the squared standard deviation (i.e., variance) of each Big Five trait was computed. Next, these variances were averaged to calculate a mean variance for each profile. Thereafter, the absolute difference between the mean variance of the respective individual's profile and the profile of their canton of residence was calculated and labeled as scatter (Furr, 2008, 2010). Thus, scatter represents a fit index that reflects the similarity in variability within profiles.

Shape was derived via a Pearson correlation on the construct level (i.e., based on the five Big Five values) between the respective individual and the corresponding canton of residence (Furr, 2008, 2010). For example, in the case of a resident of the canton of Zurich, shape similarity would be quantified by correlating the five Big Five scores of the resident of the canton of Zurich with the five aggregate Big Five scores of the canton of Zurich. Among its greatest advantages, shape (i) reflects similarity across an entire set of traits rather than averaging across them, (ii) considers trait patterns (i.e., the relative intensities of specific traits); and (iii) is not vulnerable to individual differences in scale use (De Leersnyder et al., 2011, 2014, 2015; Delvaux et al., 2015), making it the most commonly used index of profile similarity in the current framework (Furr, 2008, 2010; De Leersnyder et al., 2014).

Taken together, a good fit would be composed of high shape (high correlation and thus resemblance between the profiles under comparison), as well as low elevation (smaller distance between the profiles in question) and low scatter (less difference in variance and henceforth greater similarity).

For our main analyses, all indices were based on the construct level (i.e., five Big Five traits, rather than items). However, all indices were also calculated on the item level (i.e., ten BFI-10 items) and employed in a robustness check as reported in the Results section.

### Analysis

In keeping with the research questions outlined in the introduction, the analysis procedure is two-pronged: First, based on the aggregate Big Five traits, heat maps were created to locate and visualize the geographical distribution of personality across cantons (canton as unit of analysis). Second, multi-level modeling (MLM) was applied to investigate whether canton-level person–environment–fit in personality predicts subjective wellbeing fluctuations with individuals as unit of analysis.

## Results

### Mapping the geographical distribution of personality across switzerland

To visualize their spatial distribution across Switzerland, canton-level Big Five scores had to be calculated. To that end, every participant who had completed the BFI-10 at some point was considered. Canton of residence in the year of Big Five assessment determined the respective canton to which participants' Big Five scores were attributed. After exclusion of minors, 7,767 participants remained. As is customary in geographical psychology, the cantons' Big Five personality profiles were derived by aggregating the scores of all inhabitants of the respective cantons (Hofstede and McCrae, 2004; Gebauer et al., 2012, 2013; Rentfrow et al., 2013; De Leersnyder et al., 2014; Bleidorn et al., 2016). The resulting descriptive statistics as well as cantonal population densities (Swiss Federal Statistics Office, 2013) are exhibited in Table 1.

**Table 1:** Descriptive statistics: cantonal population density and mean big five scores.

**Canton**	***N***	**Population**	**A**	**C**	**E**	**N**	**O**
		**Density (2010)**	***M (SD)***	***M (SD)***	***M (SD)***	***M (SD)***	***M (SD)***
Aargau	705	438	6.78 (1.48)	7.39 (1.49)	6.86 (1.79)	3.39 (1.68)	6.24 (1.89)
Appenzell inner rhodes	12	91	7.33 (1.69)	7.86 (1.3)	7.84 (1.92)	2.63 (1.41)	6.33(1.78)
Appenzell outer rhodes	66	218	6.93 (1.5)	7.29 (1.41)	6.77 (1.91)	3.59 (1.72)	6.13 (2.04)
Basel-landschaft	138	530	6.95 (1.48)	7.54 (1.57)	6.8 (1.93)	3.39 (1.67)	6.59 (2.2)
Basel-stadt	289	4,999	6.78 (1.42)	7.36 (1.54)	6.78 (1.79)	3.5 (1.79)	6.43 (1.94)
Bern	1,001	168	6.98 (1.35)	7.46 (1.44)	6.87 (1.82)	3.46 (1.67)	6.18 (1.86)
Fribourg	289	175	6.77 (1.48)	7.44 (1.68)	6.19 (1.96)	3.65 (1.67)	6.29 (1.75)
Geneva	313	1,862	6.58 (1.32)	7.15 (1.46)	5.87 (1.66)	3.85 (1.77)	6.56 (1.63)
Glarus	39	57	7.23 (1.41)	7.88 (1.49)	7.59 (1.84)	3.15 (1.91)	6.33 (1.93)
Grisons	160	27	6.98 (1.49)	7.5 (1.45)	6.85 (1.81)	3.47 (1.87)	6.33 (1.95)
Jura	21	84	6.76 (1.27)	7.48 (1.87)	6.29 (2.23)	3.71 (1.86)	6.36 (2.25)
Lucerne	435	264	7.07 (1.31)	7.41 (1.49)	6.9 (1.76)	3.47 (1.65)	6.24 (1.94)
Neuchâtel	386	240	6.64 (1.36)	7.26 (1.59)	5.96 (1.98)	3.69 (1.88)	6.18 (1.77)
Nidwalden	37	170	7.22 (1.33)	7.65 (1.26)	7.46 (1.59)	3 (1.62)	6.29 (1.58)
Obwalden	31	74	6.9 (1.26)	7.45 (1.57)	7.02 (1.32)	3.35 (1.64)	5.59 (1.69)
Schaffhausen	71	256	7.12 (1.35)	7.52 (1.39)	7.29 (1.76)	3.14 (1.69)	6.46 (2.03)
Schwyz	128	172	6.58 (1.38)	7.41 (1.59)	7.19 (1.6)	3.36 (1.73)	6.23 (1.97)
Solothurn	288	323	6.85 (1.48)	7.51 (1.43)	6.89 (1.8)	3.57 (1.78)	6.15 (1.89)
St. Gallen	428	245	6.93 (1.42)	7.62 (1.48)	7.08 (1.73)	3.21 (1.74)	6.29 (1.73)
Thurgovia	185	288	6.98 (1.38)	7.51(1.57)	7.12 (1.95)	3.35 (1.73)	5.98 (1.97)
Ticino	250	122	6.59 (1.61)	6.78 (1.79)	5.66 (1.68)	3.87 (1.86)	6.34 (1.81)
Uri	18	34	7.17 (1.2)	7.75 (1.47)	6.78 (1.79)	3.61 (1.84)	6.39 (2.07)
Vaud	776	253	6.54 (1.49)	7.21 (1.67)	5.91 (1.84)	3.75 (1.75)	6.46 (1.74)
Valais	259	60	6.92 (1.39)	7.61 (1.51)	6.32 (2.03)	3.77 (1.7)	6.18 (1.83)
Zug	87	546	6.69 (1.35)	7.33 (1.43)	6.79 (1.86)	3.57 (1.63)	6.48 (1.75)
Zurich	1,355	827	6.87 (1.39)	7.3 (1.43)	6.89 (1.82)	3.43 (1.63)	6.29(1.94)

*A, Agreeableness; C, Conscientiousness; E, Extraversion; N, Neuroticism; O, Openness to experience*.

**Figure 1:**
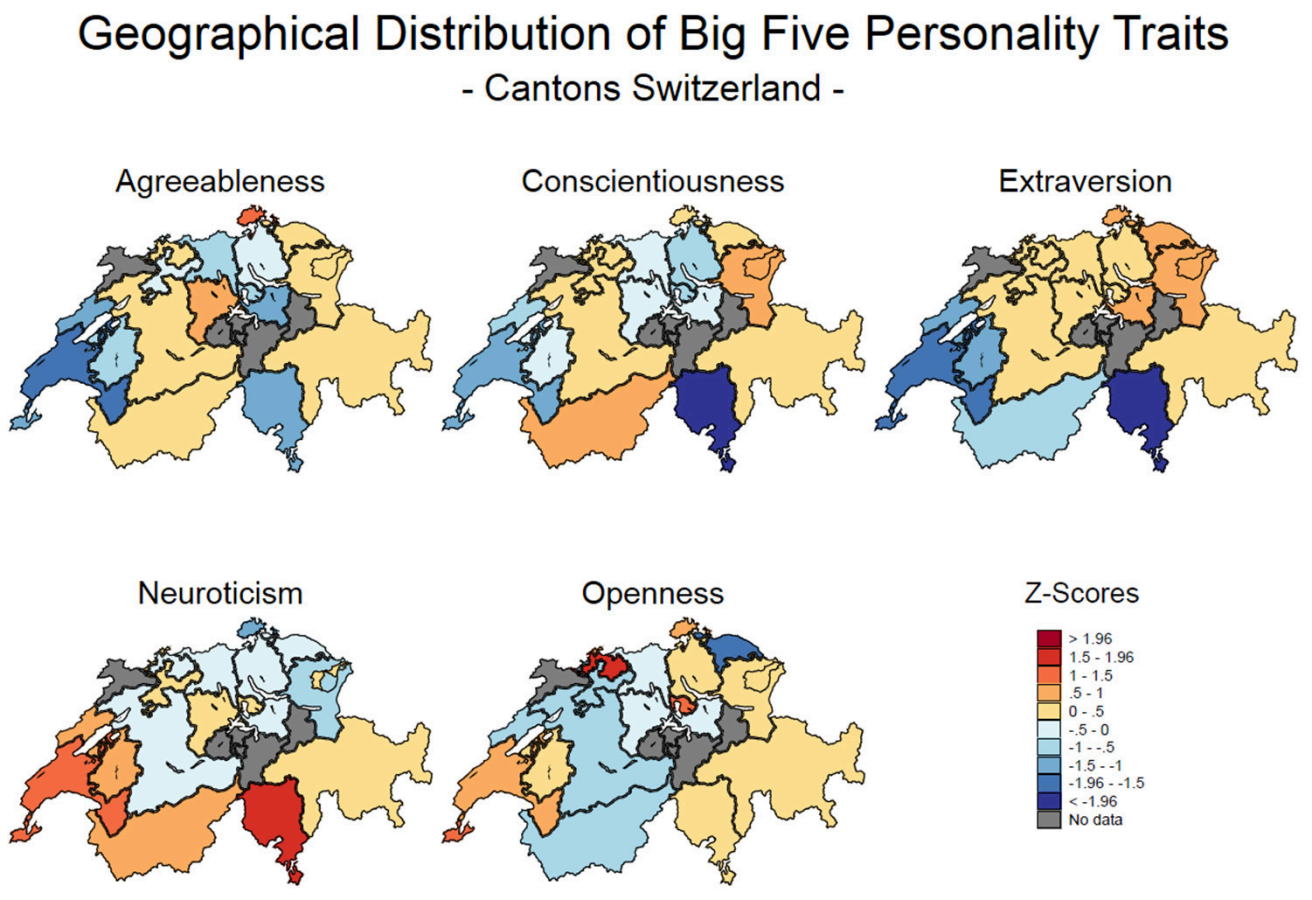
Heat maps of the geographical distribution of personality in Switzerland by canton. For each personality trait, the areas in shades of blue are comparatively low and the areas in shades of red are comparatively high.

Six cantons were excluded from analysis, as their personality means were based on <50 people (i.e., Appenzell Inner-Rhodes, Glarus, Jura, Nidwalden, Obwalden and Uri) and henceforth considered prone to bias. The maps displayed in Figure 1 were created using the **spmap** command in Stata 14.1 (Pisati, 2008). Mirroring previous research in the UK (Rentfrow et al., 2015), unique patterns emerged for the Big Five traits, although only Extraversion and Neuroticism showed clearly polarized distributions.

As can be seen in Figure 1, the spatial distribution of Agreeableness hints at a North East-South West divide. More to the point, the highest concentration of Agreeableness is found in the German-speaking North Eastern edge of Switzerland, i.e., Schaffhausen, with the canton of Lucerne in central Switzerland scoring second highest, suggesting that large portions of the residents of these areas were warm, friendly and trusting. In contrast, the lowest Agreeableness scores are detected in the westernmost, southern cantons of Vaud and Geneva, suggesting that people living in this region tended to be comparatively stubborn, demanding and quarrelsome. However, lacking conformity with a clear North East-South West divide, a few adjacent cantons in the German-speaking North of Switzerland display comparatively low prevalences of Agreeableness, i.e., Aargau, Schwyz, Solothurn, Zug, and Zurich. Similarly, the Italian-speaking Ticino also shows low rates of Agreeableness. Aside from that, the largest parts of Switzerland, ranging from the bilingual Valais to Basel-Landschaft and Grisons to Thurgovia do not form strong patterns, mostly being slightly above the national Agreeableness average.

Figure 1 further illustrates that a similar geographical distribution exists for Conscientiousness. Again, a pocket in northern Switzerland as well as the French-speaking Southwest tend to display lower levels of Conscientiousness, with the country's lowest concentration of Conscientiousness occurring in the Italian-speaking Ticino, hinting at higher shares of indifferent, rebellious and disorderly people in these regions. With most of central Switzerland once again being slightly above the national average, there are elevated levels of Conscientiousness in the southern canton of Valais as well as the eastern cantons of St. Gallen and Appenzell Outer Rhodes, suggesting that inhabitants of these areas tended to be self-disciplined, dutiful and orderly.

Whereas Agreeableness and Conscientiousness showed somewhat fragmented and inconsistent geographical patterns as outlined above, a very clear-cut divide exists for Extraversion. Hereby, Extraversion is highest among the German-speaking eastern cantons i.e., Appenzell Outer Rhodes, Schaffhausen, Schwyz, St. Gallen, Thurgovia, suggesting that large proportions of residents in this part of the country were sociable, energetic and talkative. Meanwhile, the lowest levels of Extraversion are all found in southwestern Switzerland, i.e., French-speaking Fribourg, Geneva, Neuchâtel, Valais, Vaud and the Italian-speaking Ticino, thus indicating that residents of these cantons tended to be introverted, reserved and quiet.

Clear patterns were found when mapping out geographical variation in Neuroticism, with the data producing a nearly inverse mirror image of the Extraversion map. As such, comparatively low concentrations were located in the German-speaking Eastern cantons of Appenzell Outer Rhodes, Schaffhausen, St. Gallen, suggesting that inhabitants of these areas were contented, self-confident and emotionally stable. High concentrations of Neuroticism appeared primarily in the French-speaking Western part of the country (i.e., Fribourg, Geneva, Neuchâtel, Valais, and Vaud) and the Italian-speaking Ticino, suggesting that people living in these regions were comparatively moody, tense and depressed.

Lastly, Figure 1 also exhibits the spatial distribution of Openness. Here, a more nuanced picture emerges. In the absence of a clear pattern and with the majority of cantons deviating only little from the national average, there are a few scattered, yet noteworthy pockets of elevated Openness. Those appear in Geneva and Vaud in the Southwest, Basel-Stadt and Basel-Landschaft, as well as Schaffhausen up north and Zug in central Switzerland, corresponding to higher shares of creative, curious, and unconventional residents. In contrast, the lowest Openness scores in all of Switzerland emerged in Thurgovia, speaking of a greater dominance of conventional, traditional, and down-to-earth people among the population of this canton.

### Multilevel modeling: predicting subjective wellbeing

Harnessing the longitudinal structure of the panel data, the impact of the three person–environment–fit indices on the development of various indicators of subjective wellbeing (i.e., life satisfaction, satisfaction with personal relationships, positive affect, negative affect) was examined. As it yielded the best trade-off between sample size, availability of fit indices and length of observational timeframe, 2012 (wave 14) was set as base year. For the Big Five, which are not available longitudinally, a cumulative canton-level person–environment–fit was computed with data from 2009 (wave 11) to 2011 (wave 13). Again, canton of residence in the year of Big Five assessment determined the respective canton to which participants' Big Five scores were attributed. Mirroring the foregoing mapping approach, six cantons whose personality profiles were calculated from <50 people (i.e., Appenzell Inner-Rhodes, Glarus, Jura, Nidwalden, Obwalden and Uri) were excluded from analysis. Table 2 provides a summary of the independent variables, whereas Table 3 offers descriptive statistics of the dependent variables. In keeping with the data structure in the analyses described below, Table 2 uses individual panel participants as unit of analysis, while Table 3 uses person-years, i.e., each annual observation per person, as unit of analysis.

**Table 2:** Descriptive statistics: predictors of subjective wellbeing.

	***N***	***M(SD)***	***Min***	***Max***
Big Five PE fit: elevation	6,713	0.61 (0.48)	0.01	2.99
Big Five PE fit: shape	6,713	0.67 (0.34)	−0.98	0.99
Big Five PE fit: scatter	6,713	2.58 (2.62)	0.02	18.59

*PE fit, person–environment–fit*.

**Table 3:** Descriptive statistics: longitudinal outcome measures of subjective wellbeing.

	***N***	***M(SD)***	***Min***	***Max***
Life satisfaction	27,535	7.99 (1.33)	0	10
Satisfaction with personal relationships	27,535	8.27 (1.4)	0	10
Positive affect	27,535	7.09 (1.73)	0	10
Negative affect	27,535	2.16 (2.05)	0	10

When examining within-person variability in psychological states, multilevel modeling (MLM) is usually the method of choice (Nezlek, 2008). In the present case, the multilevel structure is expressed in temporal hierarchies, i.e., repeated measures of subjective wellbeing indices (Level 1) are nested within individuals (Level 2; Nezlek, 2008; Bell and Jones, 2015). Consequently, time-varying observations are measured at Level 1, while Level 2 encompasses time-invariant, unchanging attributes such as person–environment–fit in 2011 (Bell and Jones, 2015). Then, implementing MLMs means estimating a Level 1 model (dependent measures, e.g., life satisfaction, positive affect) for every Level 2 unit (in our case every SHP participant whose data are analyzed). Adding one more layer of complexity to our model, given our research focus on regional cultures we also had to account for potential cantonal differences in the observed relationships. In conceptual equivalence to models in cross-cultural psychology (Nezlek, 2010), where individuals are nested in countries, we henceforth also nested individuals in cantons (Level 3). More specifically, to control for Level 3 (canton level) differences, we applied group-mean centering at Level 2 consistent with Nezlek's, recommendations (Nezlek, 2010).

Following Nezlek (2010), we first ran a null model which has no predictors at either level of analysis and allows to see how much of the total variance of the observed criterion is at the different levels of analysis. Calculating variance partition coefficients demonstrated that the largest shares of variance were found at the inter-individual level (Level 2; life satisfaction: 54.39%, satisfaction with personal relationships: 51.17%, positive affect: 44.22, negative affect: 58.68%) as well as intra-individual level (Level 1; life satisfaction: 44.25%, satisfaction with personal relationships: 48.31%, positive affect: 55.57%, negative affect: 39.35%), while very little variance was found at the canton level (Level 3; life satisfaction: 1.36%, satisfaction with personal relationships: 0.52%, positive affect: 0.21%, negative affect: 1.97%), which mirrors prior research (Bleidorn et al., 2016).

Turning to the test of our hypothesis, we then fitted multilevel models for all four dependent variables, using the **xtmixed** command in Stata 14.1. The results are displayed in Table 4. For easier interpretation, we standardized all variables prior to analyses and thus report standardized betas of the fixed coefficients (Nezlek, 2010) with 95% confidence intervals (Thompson, 2002). In addition, Wald-χ^2^-statistics, model degrees of freedom and *N* (Level 1) as well as *N* (Level 2) and *N* (Level 3) are presented. All models were significant (Model 1: Wald-χ^2^ = 511.39^*^; Model 2: Wald-χ^2^ = 684.39^*^; Model 3: Wald-χ^2^ = 1107.99^*^; Model 4: Wald-χ^2^ = 1405.56^*^). Moreover, in all four cases the three-level model offered a significantly better fit to the data than a single level model (Model 1: χ^2^ = 9323.74; Model 2: χ^2^ = 7718.61; Model 3: χ^2^ = 5104.96; Model 4: χ^2^ = 10389.39; Model 1–4: *df* = 2).

**Table 4:** MLM, Listing Predictors and Dependent Variables Individually.

**Predictors**	**Life satisfaction**	**Satisfaction with personal relationships**	**Positive affect**	**Negative affect**
	**Model 1**	**Model 2**	**Model 3**	**Model 4**
	Wald–*χ^2^* = 511.39^*^	Wald–*χ^2^* = 684.39^*^	Wald–*χ^2^* = 1107.99^*^	Wald–*χ^2^* = 1405.56^*^
	*df* = 6 *N* (Level 1) = 27,465	*df* = 6 *N* (Level 1) = 27,465	*df* = 6 *N* (Level 1) = 27,465	*df* = 6 *N* (Level 1) = 27,465
	*N* (Level 2) = 6,705	*N* (Level 2) = 6,705	*N* (Level 2) = 6,705	*N* (Level 2) = 6,705
	*N* (Level 3) = 20	*N* (Level 3) = 20	*N* (Level 3) = 20	*N* (Level 3) = 20
	β [95% CI]	β [95% CI]	β [95% CI]	β [95% CI]
Sex	−0.005 [−0.057, 0.048]	−0.098^*^ [−0.136, −0.061]	0.05^*^ [0.015,0.85]	−0.273^*^ [−0.311, −0.236]
Age	0.019^*^ [0.001, 0.037]	0.026^*^ [0.001, 0.044]	−0.082^*^ [−0.098, −0.065]	0.099^*^ [0.081, 0.117]
Education status	0.016 [−0.008, 0.040]	−0.086^*^ [−0.109, −0.062]	−0.002 [−0.024, 0.021]	−0.004 [−0.027, 0.019]
Big Five PE fit: elevation	−0.015 [−0.034, 0.004]	−0.001 [−0.019, 0.018]	−0.016 [−0.033, 0.001]	0.003 [−0.015, 0.022]
Big Five PE fit: shape	0.134^*^ [0.114, 0.154]	0.117^*^ [0.097, 0.136]	0.222^*^ [0.204, 0.24]	−0.235^*^ [−0.254, −0.216]
Big Five PE fit: scatter	0.137^*^ [0.117, 0.157]	0.166^*^ [0.147, 0.185]	0.127^*^ [0.109, 0.145]	−0.145^*^ [−0.164, −0.126]

* *p < 0.05, CI, confidence interval; sex, male = 1, female = 0; PE fit, person–environment–fit*.

In keeping with the guidelines assembled by Nezlek (2010) in the remainder of the results section we focus on the contributions and meaning of individual parameters rather than the overall fit of the model.

As can be seen in Table 4, sex, age and education status were each related to some positive and some negative outcomes, without producing consistent effects. Regarding our hypothesis *(higher person–environment–fit predicts increased subjective wellbeing)* mixed results were found, even though the findings for every fit index (i.e., elevation, scatter, shape) in isolation were consistent. In accordance with our assumptions Big Five shape similarity significantly predicted higher life satisfaction (β = 0.134^*^), enhanced satisfaction with personal relationships (β = 0.117^*^), stronger positive affect (β = 0.222^*^) as well as reduced negative affect (β = −0.235^*^). Meanwhile, failing to support our hypothesis, Big Five elevation did not emerge as significant predictor of subjective wellbeing. Lastly, in clear conflict with our expectations, Big Five scatter consistently predicted subjective wellbeing in the opposite direction of our predictions. As such, higher scatter, which is indicative of decreased fit, was significantly related to elevated life satisfaction (β = 0.137^*^), greater satisfaction with personal relationships (β = 0.166^*^), more positive affect (β = 0.127^*^) and less negative affect (β = −0.145^*^).

To ensure the validity of the findings, a series of robustness checks was applied. First, in recognition of the hierarchical structure of personality, McCrae (2015) has argued that nuances, which may correspond to single items, constitute a meaningful layer of personality that captures variance beyond the conventionally used Big Five facets (i.e., Agreeableness, Conscientiousness, Extraversion, Neuroticism, Openness). In accordance with this claim, several researchers in geographical psychology have run additional analyses with Big Five traits on nuance-, i.e., item-level, finding considerably smaller (Mõttus et al., 2017) or even substantially different effects (Rentfrow and Jokela, 2017). Against this backdrop, we reran all four models, computing Big Five fit indices on item-level. As can be seen in Table S1 which is available in the online supplement, the effects that were observed in the original models remained unchanged.

Second, Wood and Furr (2016) have pointed out an important methodological pitfall related to holistic fit indices, which they called the normative-desirability confound (NDC). They argue that common fit indices may systematically overestimate similarity between two individual profiles due to the fact that most of their shared variance is actually rooted in normativeness. Normativeness in turn tends to be highly confounded with the desirability of a psychological profile. Providing compelling evidence for their claim, Wood and Furr (2016) reason that the NDC can be overcome by using distinctive similarity indices. Hereby, the normativeness of each profile is removed by subtracting the average profile across individuals, although it should be kept in mind that this procedure reduces reliability and may actually remove meaningful shared variance that is not caused by the NDC (Wood and Furr, 2016). Whilst we acknowledge the relevance of the NDC, it is important to note that the context of our work might require a different approach to deal with it. Unlike Wood and Furr (2016) we do not compare two individuals but rather an individual and a norm, i.e., the aggregate personality profile of its canton of residence as an expression of regional culture. Removing the normativeness would thus erase most of the variance contained in the cantonal profiles and would in turn leave little to which the individual profiles could be compared.

We tried to account for the NDC by removing the Big Five trait that is most likely to be confounded with desirability, i.e., Neuroticism from our model. Neuroticism is evidently the least desirable of the Big Five traits (Hayes and Dunning, 1997; Smith and Ellingson, 2002; Wood and Furr, 2016). Accordingly, diminished Neuroticism has been consistently linked to perceived normality across various large-scale samples (Wood et al., 2007). Beyond that, Neuroticism has been shown to be especially prone to induce bias due to self-enhancement which is in turn a strong predictor of normativeness (Borkenau and Zaltauskas, 2009; Bollich et al., 2015). At the same time, Neuroticism has been identified as the strongest individual predictor of subjective wellbeing (Costa and McCrae, 1980; Steel and Ones, 2002; Heller et al., 2004; Rentfrow et al., 2009; McCann, 2010, 2011; Jokela et al., 2015; Mõttus et al., 2017). Taken together, it is critical to control for Neuroticism in order to ensure that actual personality fit, rather than Neuroticism *per se* would produce the observed effects.

Analogous empirically derived arguments have been made for Agreeableness which stands out among the Big Five as a particularly desirable and sought-after trait (Paulhus et al., 1995; Paulhus and John, 1998; Beer and Vazire, 2017). Moreover, unlike some of the other Big Five traits that yielded inconclusive findings, heightened Agreeableness has been robustly associated with perceived normality (Wood et al., 2007).

In addition, Agreeableness might be doubly important in the present research context, given its crucial role in what can essentially be construed as an elementary matter of fit in daily life: personal relationships. Indeed, ample empirical evidence showed that Agreeableness is a key predictor of various indicators of a good romantic match such as marital satisfaction (Botwin et al., 1997; Heller et al., 2004; Shackelford et al., 2008), sexual satisfaction (Botwin et al., 1997) but also social satisfaction (Heller et al., 2004). In fact, a recent meta-analysis identified Neuroticism and Agreeableness as the two strongest predictors of relationship satisfaction among the Big Five (Malouff et al., 2010). Thus, while Neuroticism appears to be the trait that is most likely to be confounded with desirability, additionally controlling for Agreeableness which might also be a potential confound offers an even more conservative robustness check.

To that end, we adopted a two-pronged approach whereby we first ran a model that added Neuroticism and Agreeableness as individual predictors to the existing ones (i.e., sociodemographic variables, Big Five; see Table S2). We then ran a second model, without Neuroticism in which the Big Five fit indices were calculated based on Agreeableness, Conscientiousness, Extraversion and Openness, only (see Table S3). Analogously, we ran the same model, this time without Agreeableness and henceforth with the Big Five indices being based on Conscientiousness, Extraversion, Neuroticism, and Openness, only (see Table S4). As shown in the corresponding tables of the online supplement, the findings of the original models were all replicated when Neuroticism was controlled for (see Table S2), except for Model 4 where scatter did no longer predict negative affect. Similarly, the original patterns persisted when Agreeableness was removed from the fit indices, with elevation emerging as a negative predictor of life satisfaction (β = −0.024^*^), concordant with our hypothesis (see Table S4).

However, more noteworthy deviations from the original findings occurred when removing Neuroticism from the fit indices, as becomes apparent in Table S3. Whilst elevation was once again not significantly related to any of the outcomes and only minor changes were found for shape, which was no longer a significant predictor of satisfaction with personal relationships (Model 2), a more complex picture emerged for scatter. Of note, whereas scatter was no longer predictive of life satisfaction (Model 1), as in the previous analyses it was still positively related to satisfaction with personal relationships (Model 2). More importantly, in contrast with the results that had been observed before when Neuroticism was removed from the fit indices, higher scatter was significantly associated with lower positive affect (β = −0.041^*^) and higher negative affect (β = 0.022^*^), which conforms to our original fit hypothesis.

Third and last, one might argue that the cutoff-value of *n* = 50 to include cantons in the MLM might not be conservative enough. Thus, in order to rule out that this decision may have affected our outcomes, we varied the cutoff-value and reran the model, setting a minimum cell size of 200 for inclusion in the model. As a result, the number of cantons that were included at Level 3 shrunk to 13. Again, as can be seen in Table S5, varying the sample size cutoff-value did not change the outcomes of our model, except for Model 1 (life satisfaction; β = −0.022^*^) and Model 3 (positive affect; β = −0.022^*^) where Big Five elevation emerged as significant predictor in line with our original expectations.

Taken together, the present analysis yielded mixed results with respect to our hypothesis. More specifically, the findings offered strong empirical evidence in favor of the positive impact of shape, with the effects holding up through a series of various robustness checks, thus inspiring confidence in their validity. In contrast, while the few occasions when elevation emerged as statistically significant predictor pointed in the hypothesized direction, in the vast majority of cases it yielded null findings, thus failing to provide much support for our predictions. Intriguingly, all scatter predictors produced effects in the opposite direction of those predicted, except for one robustness check, when Neuroticism was removed from the Big Five fit indices (see Table S3). In that case, a rather complicated, contradictory picture emerged, including null effects (Model 1), positive associations with subjective wellbeing (Model 2) and negative associations with subjective wellbeing (Model 3).

## Discussion

The present study employed large samples from the nationally representative SHP and demonstrated that (1) there is considerable regional variation in personality traits across the 26 cantons of Switzerland, and (2) canton-level person–environment–fit predicts various indicators of subjective wellbeing. However, the findings are not as clear and straightforward as this summary suggests. Therefore, in the following paragraphs the present results are discussed in detail.

First, exploring the geographical distribution of Big Five personality traits yielded an intriguing pattern. Agreeableness, Conscientiousness and Openness showed variation that mirrored prior research (Rentfrow et al., 2008, 2013, 2015) insofar as neighboring cantons tended to be similar, with clusters arising in the French-speaking Southwest, the Italian speaking Ticino, the easternmost German-speaking cantons and the more central areas. However, no clear overarching division emerged for these traits. Interestingly, a strong North East-South West divide, corresponding to the distribution of predominant language (German vs. French and Italian) was observed for Extraversion and Neuroticism. The French-speaking West as well as the Italian-speaking Ticino exhibited high concentrations in Neuroticism and low levels of Extraversion, whereas the German-speaking East and some parts of central Switzerland showed the opposite pattern. Intuition suggests that this may—at least in part—be due to bidirectional cultural spill-over, migration and social interaction effects (Krug and Kulhavy, 1973) with the neighboring countries (i.e., France, Germany and Italy). Indeed, the findings of a large-scale cross-national investigation that collected Big Five data across 56 nations (Schmitt et al., 2007) appear to back up this notion. In line with the idea of spill-over effects, average neuroticism levels in France (*M* = 52.29, *SD* = 9.34) and Italy (*M* = 57.87, *SD* = 7.38) were higher than in Germany (*M* = 50.29, *SD* = 8.44). Likewise, the reversed pattern was found for Extraversion which was considerably higher in Germany (*M* = 50.31, *SD* = 8.99) than in France (*M* = 45.44, *SD* = 8.77) and marginally higher than in Italy (*M* = 49.80, *SD* = 8.09). This finding hints at potentially meaningful cultural interaction effects in border regions and offers an interesting avenue for future research.

Moving on to the focal investigation of the present research, i.e., the effects of person–environment–fit on the development of subjective wellbeing mixed, results occurred. We attempted to measure fit holistically, using three components, i.e., elevation, scatter and shape, that were expected to produce converging results (Furr, 2008, 2010; Wood and Furr, 2016). Instead, each fit index produced a unique pattern that was consistent across all outcomes (i.e., life satisfaction, satisfaction with personal relationships, positive affect, negative affect). Indeed, higher shape predicted increased subjective wellbeing throughout all analyses. With respect to elevation, despite some significant findings in the robustness checks that were in line with our expectations, only null findings occurred in the main analyses. Even more extreme, higher scatter was related to elevated subjective wellbeing, thereby contradicting our assumptions. However, while this pattern persisted for most robustness checks, results that were predictive of lower subjective wellbeing (and hence conforming to our hypothesis) were observed for two of the four outcome variables when Neuroticism was removed from the Big Five fit indices to account for normativeness and desirability (Wood and Furr, 2016).

In trying to reconcile these divergent findings, it might be fruitful to seek clarification in the management literature, where person–environment–fit has been studied for much longer than in any other discipline (Parsons, 1909; Jansen and Kristof-Brown, 2006). Here, scholars have questioned what has commonly been treated as a given in fit research: that one needs to be similar to one's environment to fit in (Kristof, 1996). At times, the opposite may actually be true and deviation may be beneficial and help to fill a gap or compensate for a weakness in one's environment (Piasentin and Chapman, 2006). Owing to this observation, Muchinsky and Monahan (1987) coined the terms supplementary and complementary fit. Whereas, the former captures the widespread notion of fit as a concept of sameness, the latter construes fit as the positive result of mutually offsetting profiles (Muchinsky and Monahan, 1987), very much like folk wisdom that knows that “birds of a feather flock together” just like “opposites attract”. Thus, as contradictory as it sounds at first, the idea of complementary fit or fitting in by being different appears quite plausible and dovetails well with other conceptual frameworks such as optimal distinctiveness theory (Brewer, 2012). Likwise, the theory of uniqueness (Snyder and Fromkin, 1980) posits that people's need for uniqueness drives them to want to stand out from their surroundings to boost their self-esteem and intrinsic satisfaction. Yet in spite of its great explanatory potential, the concept of complementary fit has never fully entered the mainstream of organizational psychology and is thus still underexplored (Kristof, 1996).

Nevertheless, looking through this lens may shed new light on the present findings. Indeed, as Furr (2010) pointed out, elevation, scatter and shape are in fact independent indicators that tap into different elements of fit and do not necessarily yield the same results. In light of this argument, the current results offer a fresh, yet very preliminary new perspective on holistic fit. Therein scatter might be measuring complementary fit, while shape would assess supplementary fit. Given the consistency of our findings across four different indicators of subjective wellbeing and various robustness checks the role of shape as an index of supplementary fit might be assumed with some confidence. However, much caution is warranted with respect to scatter. Encouragingly, the majority of our results would support the proposed classification of scatter as an index of complementary fit. Still, the mixed results shown in Table S3 suggest that the impact of scatter might be sensitive to concrete modeling and operationalization. Consequently, it must be stressed that the current research should only be construed as a very first step toward such a new conceptualization of different facets of person–environment–fit. To further advance this theory of different fit facets, extensive empirical tests are imperative. That way, converging evidence might be accumulated that would be theory-driven and hence not subject to claims of reverse inference.

Similarly, the contribution of elevation that did not seem particularly important in the given context needs to be subject to further scrutiny. In this vein, it should be noted that although it is customary to use absolute values to assess elevation between profiles (e.g., Ward and Chang, 1997; Chirkov et al., 2005; Kashima and Abu-Rayya, 2014), this practice may not be ideal. When using absolute values, positive and negative deviations do not cancel each other out, but are accumulated irrespective of their direction. While this procedure thus provides reliable estimates about the magnitude of differences, it obscures their direction, thereby arguably missing a critical piece of information (Lu, 2006). As scatter and shape are calculated differently, they are not affected by this potential bias. This might be one of the reasons why both scatter and shape emerged as important predictors of subjective wellbeing in the present study, whereas elevation did not.

Pending successful replication and consolidation, the importance of the present pattern of results should not be overestimated. Nevertheless, the findings suggest that it might be useful to venture beyond mere profile correlations as the most widely used, easily accessible and arguably most relevant of the three fit indices (Furr, 2010). Instead adopting a holistic perspective might be informative, although more needs to be learned about scatter and elevation to fully leverage their potential and understand their consequences.

At the same time, the notion of supplementary vs. complementary fit and optimal distinctiveness (Brewer, 2012) may not only be explored across, but also within different facets of fit. Specifically, future research might benefit from considering the possibility of non-linear fit effects, where thresholds of optimal distinctiveness might be identified. As a preliminary step in this direction, we re-ran our main analyses, including quadratic terms to account for potential non-linear effects. Indeed, with the exception of relationship satisfaction which did not yield any quadratic effects, a consistent picture emerged. Therein, no quadratic effects were found for scatter, whereas the results for both shape (life satisfaction: β = 0.022^*^; positive affect: β = 0.028^*^; negative affect: β = −0.04^*^) and elevation (life satisfaction: β = −0.014^*^; positive affect: β = −0.012^*^; negative affect: β = 0.015^*^) pointed to the existence of non-linear effects. Although preliminary these findings suggest that fit is not necessarily a matter of sameness and deserves increased attention as it appears to be a much more complex phenomenon than previously assumed.

In light of these considerations, we tentatively draw the following conclusions: First, the study of cultural differences at the regional level offers a fruitful addition to traditional nation-based approaches to culture (Rentfrow and Jokela, 2016), with the present study attesting to the relevance and far-reaching consequences of said cultural differences.

Second, person–environment–fit is a highly relevant concept within and beyond geographical psychology that can offer incremental value to the study of key conceptual issues such as changes in subjective wellbeing.

Third, in line with Furr (2008, 2010) and Wood and Furr (2016), the present study demonstrates advantages of a differentiated assessment of person–environment–fit over omnibus indices, thus offering a more nuanced understanding of the underlying dynamics of the processes under study. At the same time, it yields some puzzling findings mainly with regards to scatter that call for increased research efforts to foster the understanding of the components of person–environment–fit and their joint and individual influences. In this vein, whilst shape conforms to the traditional idea of fit as a concept of sameness (i.e., supplementary fit), future research is needed to illuminate whether, and if so under which circumstances scatter may be an index of complementary fit. Also, some limitations of the present work should be noted.

### Limitations

Although the 26 Swiss cantons offer a higher geographical resolution than, for instance the 50 American states, they may still be too coarse to adequately capture the fit with people's actual primary living environments and local culture. More fine-grained levels such as cities (Park and Peterson, 2010) or neighborhoods (Jokela et al., 2015) may be even more informative, yield even stronger fit effects (Stavrova and Luhmann, 2016) and extend the understanding of culture to the local level.

Moreover, given the multilingual structure of Switzerland, the SHP is administered in four different languages (i.e., English, French, German, Italian). Although great care was taken by the responsible researchers at the Swiss Centre of Expertise in the Social Sciences FORS to ensure equivalence by using established measures with solid psychometric properties and thoroughly validated translations whenever possible, one might raise concerns about measurement invariance. However, prior research in cultural psychology has undertaken extensive comparisons across various multilingual cohorts (e.g., in Hong Kong, Zimbabwe and Hispanic Americans; McCrae, 2001), thoroughly demonstrating that multilingual Big Five questionnaires do not introduce bias (Allik and McCrae, 2004; McCrae et al., 2005).

Furthermore, due to the data structure of the SHP, person–environment–fit in Big Five personality traits was not available on a longitudinal level and hence could not vary over time along with the predicted outcomes. However, we argue that it appears reasonable to assume that both individual personality and average canton-level personality remain fairly stable and henceforth time-invariant over the timeframe under observation. Recognizing that personality has been shown to be prone to change at an early age while remaining rather stable and consistent across adulthood (McGue et al., 1993; Roberts and DelVecchio, 2000; Soto et al., 2011), we excluded all minors of <18 years from analysis. Further supporting our claim, the empirically derived corresponsive principle holds that personality trait development that does occur in adulthood primarily deepens existing characteristics (Caspi et al., 2005). Thus, if anything, personality change during the timeframe of our investigation would have most likely only amplified the individual fit tendencies (i.e., further increasing good fit and exacerbating bad fit). Meanwhile, spanning across four decades, four different studies on regional personality differences in the United States yielded strongly convergent results (Rentfrow et al., 2008), thus demonstrating a high degree of stability over time.

Also, it should be acknowledged that we did not account for inter-cantonal residential mobility. However, when examining the cases where the canton of residence had changed, irrespective of missing observations that would have concealed the exact time of the move, it appeared that such moving behavior was a rather rare occurrence among the sample population (wave 11 (2009): 8.57%, wave 12 (2010): 6.87%, wave 13 (2011): 6.39%, wave 14 (2012): 7.84%, wave 15 (2013): 5.83%, wave 16 (2014): 5.48%, wave 17 (2015): 5.85%). This finding is consistent with representative statistics for Switzerland as a whole, where 75% of all intra-national residential mobility takes place within the same canton (Swiss Federal Statistics Office, 2016).

Lastly, one might question the psychometric properties of some of the measures employed. Although the BFI-10 has been shown to exhibit satisfying psychometric properties (Gerlitz and Schupp, 2005; Lang, 2005; Rammstedt and John, 2007; Donnellan and Lucas, 2008; Lang et al., 2011) and has been successfully used in personality research, featuring SHP data (Anusic et al., 2012; Furler et al., 2013), inter-item correlations and reliability estimates tended to be at the lower boundaries of recommended values (Clark and Watson, 1995), leading to criticism being leveled against their use (Ryser, 2015). It should also be noted that some of the constructs in the present study were assessed with single item scales which is likely to impose further limitations on the generalizability of the present findings.

## Concluding remarks

Extending previous nation-level research to the regional level, the present work demonstrates the importance of regional cultures and the potential for joint discovery and cross-fertilization that lies at the intersection of geographical and cultural psychology. More specifically, applying state-of-the-art analysis procedures (MLM, Nezlek, 2001, 2007, 2008, 2010) to a nationally representative panel with rich longitudinal data, the current study is the first to map the regional distribution of personality across Switzerland. So far, similar psychological topographies have been observed only for large (USA: 322 m inhabitants; Rentfrow et al., 2008; Rentfrow, 2010) or medium-sized (UK: 65 m inhabitants; Rentfrow et al., 2015) countries. Switzerland is not only the first small country (8 m inhabitants) to be mapped, but also the first to be officially multilingual and located in continental Europe.

Furthermore, our research elucidates the long-term consequences of person–environment–fit on subjective wellbeing, extending previous research by measuring both person–environment–fit (Furr, 2008, 2010; Wood and Furr, 2016) and subjective wellbeing (Diener, 2012; Diener et al., 2017) in a multi-faceted and holistic manner. In view of the divergent effects of the three fit components in the present study, future research should be geared toward further consolidation and explore the applicability of qualitatively different kinds of fit, such as supplementary vs. complementary fit, to the concept of person–culture–fit.

## Ethics statement

The present study used a pre-collected panel data set from the Swiss Household Panel (SHP). Said use for the current research has been explicitly approved by the Swiss Centre of Expertise in the Social Sciences (FORS), after ethical and legal conditions had been met by our research team upon signing a bilateral contract in accordance with the data distribution policy of FORS and the SHP. The Swiss Household Panel itself was carried out in accordance with the recommendations of the Research Commission of the University of Neuchâtel. The protocol was approved by the Research Commission of the University of Neuchâtel. All subjects had been provided with written informed consent in accordance with the Declaration of Helsinki and also gave verbal consent as the data collection was conducted via telephone interviews.

## Author contributions

FG and PR: contributed conception and design of the study; FG: organized the database; TE and FG: performed the statistical analysis; FG: wrote the first draft of the manuscript; TE and PR: critically revised the manuscript. All authors have read and approved the final manuscript.

### Conflict of interest statement

The authors declare that the research was conducted in the absence of any commercial or financial relationships that could be construed as a potential conflict of interest.
