# Supplementary material for: Regional Cultures and the Psychological Geography of Switzerland: Person–Environment–Fit in Personality Predicts Subjective Wellbeing
Source: Front Psychol. 2018 Apr 16;9:517. doi: 10.3389/fpsyg.2018.00517 (PMC5911505; doi:10.3389/fpsyg.2018.00517)
Supplement: Supplementary file 1 [file Table1.docx]

**Online Supplement**

**Table S1.** *Results from the Multilevel Model with Big Five Fit Indices on Nuance- / Item-Level, Listing Predictors and Dependent Variables Individually.*

| Predictors | Life satisfaction  Model 1  Wald−*χ^^ *  = 554.97*  *df* = 6  *N* (Level 1) = 27,465  *N* (Level 2) = 6,705  *N* (Level 3) = 20 | Satisfaction with personal  relationships  Model 2  Wald−*χ^^*  = 757.88*  *df* = 6  *N* (Level 1) = 27,465  *N* (Level 2) = 6,705  *N* (Level 3) = 20 | Positive Affect  Model 3  Wald−*χ^^*  = 1139.68*  *df* = 6  *N* (Level 1) = 27,465  *N* (Level 2) = 6,705  *N* (Level 3) = 20 | Negative Affect  Model 4  Wald−*χ^^ *  =1263.3*  *df* = 6  *N* (Level 1) = 27,465  *N* (Level 2) = 6,705  *N* (Level 3) = 20 |
| --- | --- | --- | --- | --- |
|  | *β* [95% CI] | *β* [95% CI] | *β* [95% CI] | *β* [95% CI] |
| Sex | -0.022  [-0.061, 0.017] | -0.117*  [-0.153, -0.078] | 0.032  [-0.003,0.067] | -0.256*  [-0.294, -0.219] |
| Age | 0.019*  [0.001, 0.037] | 0.024*  [0.006, 0.042] | -0.08*  [-0.097, -0.064] | 0.095*  [0.077, 0.113] |
| Education  status | 0.026*  [0.002, 0.051] | -0.07*  [-0.094, -0.046] | 0.009  [-0.014, 0.031] | -0.013  [-0.037, 0.01] |
| Big Five PE fit: elevation | -0.008  [-0.027, 0.011] | 0.004  [-0.014, 0.023] | -0.007  [-0.024, 0.01] | -0.006  [-0.025, 0.012] |
| Big Five PE fit: shape | 0.162*  [0.143, 0.182] | 0.14*  [0.121, 0.159] | 0.242*  [0.224, 0.259] | -0.243*  [-0.262, -0.224] |
| Big Five PE fit: scatter | 0.131*  [0.111, 0.15] | 0.172*  [0.154, 0.191] | 0.121*  [0.103, 0.139] | -0.116*  [-0.134, -0.097] |

*Note*. * *p* < .05, CI = confidence interval, sex: male = 1, female = 0, PE fit = person–environment–fit.

**Table S2.** *Results from the Multilevel Model Controlling for Neuroticism and Agreeableness, Listing Predictors and Dependent Variables Individually.*

| Predictors | Life satisfaction  Model 1  Wald−*χ^^ *  = 573.00*  *df* = 8  *N* (Level 1) = 27,465  *N* (Level 2) = 6,705  *N* (Level 2) = 20 | Satisfaction with personal  relationships  Model 2  Wald−*χ^^*  = 750.27*  *df* = 8  *N* (Level 1) = 27,465  *N* (Level 2) = 6,705  *N* (Level 2) = 20 | Positive Affect  Model 3  Wald−*χ^^*  = 1156.02*  *df* = 8  *N* (Level 1) = 27,465  *N* (Level 2) = 6,705  *N* (Level 2) = 20 | Negative Affect  Model 4  Wald−*χ^^ *  = 1593.63*  *df* = 8  *N* (Level 1) = 27,465  *N* (Level 2) = 6,705  *N* (Level 2) = 20 |
| --- | --- | --- | --- | --- |
|  | *β* [95% CI] | *β* [95% CI] | *β* [95% CI] | *β* [95% CI] |
| Sex | -0.015  [-0.055, 0.024] | -0.092*  [-0.129, -0.054] | 0.039*  [0.003,0.074] | -0.239*  [-0.276, -0.201] |
| Age | 0.018  [-0.001, 0.037] | 0.021*  [0.004, 0.039] | -0.081*  [-0.098, -0.064] | 0.094*  [0.077, 0.111] |
| Education  status | 0.017  [-0.008, 0.041] | -0.085*  [-0.109, -0.062] | -0.001  [-0.023, 0.022] | -0.005  [-0.028, 0.018] |
| Big Five PE fit: elevation | -0.016  [-0.035, 0.003] | -0.004  [-0.023, 0.014] | -0.016  [-0.033, 0.001] | 0.001  [-0.017, 0.019] |
| Big Five PE fit: shape | 0.055*  [0.022, 0.087] | 0.09*  [0.059, 0.122] | 0.155*  [0.126, 0.184] | -0.08*  [-0.112, -0.049] |
| Big Five PE fit: scatter | 0.068*  [0.039, 0.097] | 0.138*  [0.11, 0.166] | 0.069*  [0.043, 0.095] | -0.016  [-0.044, 0.011] |
| Neuroticism | -0.057*  [-0.079, -0.034] | -0.004  [-0.026, 0.018] | -0.052*  [-0.072, -0.031] | 0.134*  [0.112, 0.156] |
| Agreeableness | 0.056*  [0.039, 0.071] | 0.058*  [0.043, 0.074] | 0.037*  [0.023, 0.051] | -0.047*  [-0.062, -0.032] |

*Note*. * *p* < .05, CI = confidence interval, sex: male = 1, female = 0, PE fit = person–environment–fit.

**Table S3.** *Results from the Multilevel Model with Big Five Fit Indices Computed Based on Agreeableness, Conscientiousness, Extraversion and Openness, Listing Predictors and Dependent Variables Individually.*

| Predictors | Life satisfaction  Model 1  Wald−*χ^^ *  = 36.67*  *df* = 6  *N* (Level 1) = 27,465  *N* (Level 2) = 6,705  *N* (Level 3) = 20 | Satisfaction with personal  relationships  Model 2  Wald−*χ^^*  = 113.83*  *df* = 6  *N* (Level 1) = 27,465  *N* (Level 2) = 6,705  *N* (Level 3) = 20 | Positive Affect  Model 3  Wald−*χ^^*  = 97.79*  *df* = 6  *N* (Level 1) = 27,465  *N* (Level 2) = 6,705  *N* (Level 3) = 20 | Negative Affect  Model 4  Wald−*χ^^ *  = 348.27*  *df* = 6  *N* (Level 1) = 27,465  *N* (Level 2) = 6,705  *N* (Level 3) = 20 |
| --- | --- | --- | --- | --- |
|  | *β* [95% CI] | *β* [95% CI] | *β* [95% CI] | *β* [95% CI] |
| Sex | 0.006  [-0.034, 0.046] | -0.087*  [-0.126, -0.048] | 0.068*  [0.031,0.106] | -0.292*  [-0.332, -0.252] |
| Age | 0.033*  [0.014, 0.052] | 0.042*  [0.024, 0.061] | -0.062*  [-0.079, -0.044] | 0.083*  [0.064, 0.102] |
| Education  status | 0.023  [-0.002, 0.049] | -0.078*  [-0.103, -0.053] | 0.008  [-0.016, 0.032] | -0.017  [-0.042, 0.008] |
| Big Five PE fit: elevation | 0.006  [-0.014, 0.026] | 0.035  [-0.014, 0.083] | -0.014  [-0.033, 0.004] | -0.009  [-0.028, 0.011] |
| Big Five PE fit: shape | 0.041*  [0.021, 0.061] | 0.035  [-0.006, 0.033] | 0.048*  [0.029, 0.067] | -0.077*  [-0.097, -0.057] |
| Big Five PE fit: scatter | 0.002  [-0.017, 0.022] | 0.032*  [0.013, 0.052] | -0.041*  [-0.059, -0.022] | 0.022*  [0.003, 0.042] |

*Note*. * *p* < .05, CI = confidence interval, sex: male = 1, female = 0, PE fit = person–environment–fit.

**Table S4.** *Results from the Multilevel Model with Big Five Fit Indices Computed Based on Conscientiousness, Extraversion, Neuroticism and Openness, Listing Predictors and Dependent Variables Individually.*

| Predictors | Life satisfaction  Model 1  Wald−*χ^^ *  = 474.87*  *df* = 6  *N* (Level 1) = 27,436  *N* (Level 2) = 6,699  *N* (Level 3) = 20 | Satisfaction with personal  relationships  Model 2  Wald−*χ^^*  = 623.37*  *df* = 6  *N* (Level 1) = 27,436  *N* (Level 2) = 6,699  *N* (Level 3) = 20 | Positive Affect  Model 3  Wald−*χ^^*  = 1041.81*  *df* = 6  *N* (Level 1) = 27,436  *N* (Level 2) = 6,699  *N* (Level 3) = 20 | Negative Affect  Model 4  Wald−*χ^^ *  = 1280.05*  *df* = 6  *N* (Level 1) = 27,436  *N* (Level 2) = 6,699  *N* (Level 3) = 20 |
| --- | --- | --- | --- | --- |
|  | *β* [95% CI] | *β* [95% CI] | *β* [95% CI] | *β* [95% CI] |
| Sex | 0.001  [-0.039, 0.039] | -0.092*  [-0.129, -0.054] | 0.057*  [0.022,0.092] | -0.281*  [-0.319, -0.244] |
| Age | 0.022*  [0.003, 0.04] | 0.029*  [0.011, 0.047] | -0.079*  [-0.097, -0.063] | 0.096*  [0.078, 0.114] |
| Education  status | 0.015  [-0.009, 0.039] | -0.088*  [-0.112, -0.064] | -0.003  [-0.025, 0.019] | 0.003  [-0.026, 0.021] |
| Big Five PE fit: elevation | -0.024*  [-0.043, -0.004] | 0.006  [-0.013, 0.024] | -0.019  [-0.037, -0.002] | 0.003  [-0.015, 0.022] |
| Big Five PE fit: shape | 0.119*  [0.099, 0.138] | 0.102*  [0.083, 0.122] | 0.206*  [0.188, 0.224] | -0.215*  [-0.234, -0.196] |
| Big Five PE fit: scatter | 0.143*  [0.123, 0.163] | 0.167*  [0.147, 0.186] | 0.139*  [0.122, 0.157] | -0.151*  [-0.17, -0.132] |

*Note*. * *p* < .05, CI = confidence interval, sex: male = 1, female = 0, PE fit = person–environment–fit.

**Table S5.** *Results from the Multilevel Model with Higher Canton-Level Inclusion Threshold (N>200), Listing Predictors and Dependent Variables Individually.*

| Predictors | Life satisfaction  Model 1  Wald−*χ^^ *  = 448.19*  *df* = 6  *N* (Level 1) = 24,583  *N* (Level 2) = 5,991  *N* (Level 3) = 13 | Satisfaction with personal  relationships  Model 2  Wald−*χ^^*  = 583.55*  *df* = 6  *N* (Level 1) = 24,583  *N* (Level 2) = 5,991  *N* (Level 3) = 13 | Positive Affect  Model 3  Wald−*χ^^*  = 999.86*  *df* = 6  *N* (Level 1) = 24,583  *N* (Level 2) = 5,991  *N* (Level 3) = 13 | Negative Affect  Model 4  Wald−*χ^^ *  = 1260.87*  *df* = 6  *N* (Level 1) = 24,583  *N* (Level 2) = 5,991  *N* (Level 3) = 13 |
| --- | --- | --- | --- | --- |
|  | *β* [95% CI] | *β* [95% CI] | *β* [95% CI] | *β* [95% CI] |
| Sex | -0.008  [-0.049, 0.033] | -0.094*  [-0.134, -0.054] | 0.044*  [0.007,0.081] | -0.279*  [-0.318, -0.239] |
| Age | 0.012  [-0.007, 0.032] | 0.018  [-0.001, 0.037] | -0.087*  [-0.105, -0.069] | 0.099*  [0.081, 0.119] |
| Education  status | 0.015  [-0.011, 0.041] | -0.082*  [-0.107, -0.056] | -0.004  [-0.027, 0.019] | -0.007  [-0.032, 0.018] |
| Big Five PE fit: elevation | -0.022*  [-0.042, -0.001] | -0.005  [-0.025, 0.014] | -0.022*  [-0.04, -0.004] | 0.005  [-0.015, 0.024] |
| Big Five PE fit: shape | 0.132*  [0.111, 0.154] | 0.118*  [0.097, 0.139] | 0.221*  [0.202, 0.239] | -0.235*  [-0.255, -0.214] |
| Big Five PE fit: scatter | 0.138*  [0.117, 0.159] | 0.163*  [0.143 0.184] | 0.127*  [0.108, 0.146] | -0.143*  [-0.163, -0.123] |

*Note*. * *p* < .05, CI = confidence interval, sex: male = 1, female = 0, PE fit = person–environment–fit.
